# Efficacy and Safety Analysis of Combination Therapy Consisting of Intravenous Immunoglobulin and Corticosteroids versus Respective Monotherapies in the Treatment of Relapsed ITP in Adults

**DOI:** 10.1055/s-0043-1769087

**Published:** 2023-05-23

**Authors:** Lijun Fang, Jing Sun, Yongqiang Zhao, Ming Hou, Depei Wu, Yunfei Chen, Renchi Yang, Lei Zhang

**Affiliations:** 1 State Key Laboratory of Experimental Hematology, National Clinical Research Center for Blood Diseases, Haihe Laboratory of Cell Ecosystem, Institute of Hematology & Blood Diseases Hospital, Chinese Academy of Medical Sciences & Peking Union Medical College, Tianjin, People's Republic of China; 2 Nanfang Hospital, Southern Medical University, Guangzhou, People's Republic of China; 3 Peking Union Medical College Hospital, Beijing, People's Republic of China; 4 Qilu Hospital, Cheeloo College of Medicine, Shandong University, Jinan, People's Republic of China; 5 The First Affiliated Hospital of Soochow University, Suzhou, People's Republic of China; 6 Tianjin Institutes of Health Science, Tianjin, People's Republic of China

**Keywords:** primary immune thrombocytopenia, adult relapse, first-line drugs, combination therapy

## Abstract

**Objective** In this study, we aimed to evaluate the efficacy and safety of combination therapy, consisting of intravenous immunoglobulin (IVIg) and corticosteroids, in comparison to respective monotherapies in the treatment of relapsed immune thrombocytopenia (ITP) in adults.

**Methods** A retrospective analysis of clinical data was conducted on 205 adult patients with relapsed ITP who received first-line combination therapy or monotherapy in multiple centers across China from January 2010 to December 2022. The study evaluated the patients' clinical characteristics, efficacy, and safety.

**Results** We found that the proportion of patients with platelet counts in complete response was significantly higher in the combination group (71.83%) compared with the IVIg group (43.48%) and the corticosteroids group (23.08%). The mean PLT _max_ in the combination group (178 × 10 ^9^ /L) was significantly higher than that in the IVIg group (109 × 10 ^9^ /L) and the corticosteroids group (76 × 10 ^9^ /L). Additionally, the average time for platelet counts to reach 30 × 10 ^9^ /L, 50 × 10 ^9^ /L, and 100 × 10 ^9^ /L in the combination group was significantly shorter than in the monotherapy groups. The proportion curves for reaching these platelet counts during treatment were also significantly different from those in the monotherapy groups. However, there were no significant differences in the effective rate, clinical characteristics, and adverse events among the three groups.

**Conclusion** We concluded that combining IVIg and corticosteroids was a more effective and faster treatment for relapsed ITP in adults than using either therapy alone. The findings of this study provided clinical evidence and reference for the use of first-line combination therapy in the treatment of relapsed ITP in adults.

## Introduction

Primary immune thrombocytopenia (ITP) is an acquired autoimmune disease characterized by increased platelet destruction and/or decreased platelet production. The main clinical manifestations are mucocutaneous bleeding, bleeding of vital organs, or asymptomatic thrombocytopenia. Some patients may also experience symptoms, such as fatigue and anxiety, and the clinical presentation can vary greatly among different patients.

The first-line treatment for this disease is corticosteroids and intravenous immunoglobulin (IVIg), which has a response rate of approximately 80% in newly diagnosed patients. 1 Approximately 40 to 60% of patients with newly diagnosed ITP achieve remission lasting longer than 6 months after first-line treatment, and a small proportion of patients experience remission lasting over 1 year. However, the majority of patients will experience a chronic or relapsing/remitting course. 2

There are limited clinical studies available on drug selection and efficacy evaluation for patients with chronic or persistent ITP who experience relapse, particularly those with a platelet count < 20 × 10 ^9^ /L or < 30 × 10 ^9^ /L with bleeding symptoms. Thus, this study aimed to conduct a multicenter retrospective analysis of first-line drug treatment for relapsed patients, with a specific focus on evaluating the efficacy and safety of combination therapy, involving IVIg and corticosteroids, compared with respective monotherapies in treating recurrent ITP in adults.

## Materials and Methods

### Study Design

We conducted an analysis of persistent or chronically recurrent adult ITP patients who received first-line treatment, including IVIg and/or corticosteroids, at multiple clinical centers across the country. Data were retrospectively collected from the medical files of the patients. The study protocol was approved by the medical research ethics committee at our hospital and was conducted in accordance with the ethical guidelines of the 1975 Declaration of Helsinki.

### Patients

We reviewed the medical records of patients with recurrent ITP from multiple centers between January 2010 and December 2022. Inclusion criteria were as follows: patients between 18 and 70 years old of both sexes; patients with persistent or chronically recurrent ITP who met the diagnostic criteria for ITP; platelet count less than 20 × 10 ^9^ /L, or platelet count less than 30 × 10 ^9^ /L with significant bleeding of skin, mucosa, and/or viscera; and treatment with glucocorticoid and/or IVIg for at least 7 days. Exclusion criteria were as follows: platelet transfusion within 1 week before treatment; use of other drugs for ITP treatment within 30 days before treatment, such as danazol, cyclophosphamide, vincristine, cyclosporine, recombinant human thrombopoietin (TPO)/TPO-receptor agonist (TPO-RA), interleukin-11, rituximab, IVIg, or glucocorticoids; organ dysfunction, defined as serum creatinine greater than the upper limit of normal value, alanine aminotransferase (ALT), aspartate aminotransferase (AST), and bilirubin greater than 1.2 times the upper limit of normal value; and patients after splenectomy.

### Treatment

Patients were classified into three groups based on their treatment regimen: corticosteroids group, IVIg group, and combination group (IVIg and corticosteroids). The corticosteroids group received prednisone or an equivalent glucocorticoid at a dose of 1 mg/kg/day for a minimum of seven consecutive days. The IVIg group received a continuous infusion of 5% IVIg at a dose of 0.4 g/kg/day for 5 days. The combination group received a continuous infusion of 5% IVIg at a dose of 0.4 g/kg/day for 5 days, along with prednisone or an equivalent glucocorticoid at a dose of 1 mg/kg/day for a minimum of 7 days. 5% IVIg were provided by CHENGDU RONGSHENG PHARMACEUTICALS CO. LTD.

### Outcome Measures

#### Platelet Count and Response Rate Statistics of Patients after Treatment

The platelet count changes over time during treatment (day 1–7 after the first treatment) and the maximum platelet count (PLT _max_ ) during treatment (day 1–7) were recorded for each group. Additionally, the percentage of patients reaching platelet counts of 30 × 10 ^9^ /L, 50 × 10 ^9^ /L, and 100 × 10 ^9^ /L after 7 days of treatment, as well as the proportion of complete response (CR) and response (R) after 7 days of treatment in each group, were determined. CR was defined as a platelet count of ≥ 100 × 10 ^9^ /L and no bleeding after treatment. R was defined as a platelet count of ≥ 30 × 10 ^9^ /L after treatment, which was at least two times higher than the basal platelet count, and no bleeding manifestation was present.

#### Relevant Statistics of the Onset Time of Patients

The time at which the platelet count first reached PLT _max_ during the course of treatment was recorded. Additionally, the corresponding time at which the platelet count first reached 30 × 10 ^9^ /L, 50 × 10 ^9^ /L, and 100 × 10 ^9^ /L, as well as the percentage of platelet counts reaching 30 × 10 ^9^ /L, 50 × 10 ^9^ /L, and 100 × 10 ^9^ /L each day in each group were determined.

#### Adverse Event Statistics

Adverse events (AEs) occurring during the treatment period in each group were counted.

### Statistical Analysis

Statistical analysis and graphs were generated using SPSS 22.0 software and GraphPad Prism 8.3.0. Clinical characteristics, such as general data, baseline levels of platelet count and bleeding score, medical history, and previous treatment, were expressed as mean ± standard deviation (x ® ± SD). The H test was used to compare the three groups, and independent sample *t* -tests were used for pairwise comparisons between the groups. Percentages (%) were compared between groups using the chi-squared test. The number of patients with platelet counts reaching 30 × 10 ^9^ /L, 50 × 10 ^9^ /L, and 100 × 10 ^9^ /L every day was calculated using the Kaplan–Meier method, and percentage curves of platelet counts reaching these thresholds every day in each group were drawn. Differences between groups were compared using the log-rank test. All tests were two-sided, and *p* < 0.05 was considered statistically significant. *p* -Values less than 0.05 were denoted by *, *p* -values less than 0.01 were denoted by **, *p* -values less than 0.001 were denoted by ***, and *p* -values less than 0.0001 were denoted by ****.

## Results

### Baseline Characteristics

A total of 205 patients were included in this study, with 65 patients (31.71%) in the corticosteroids group, 69 patients (33.66%) in the IVIg group, and 71 patients (34.63%) in the combination group. The age, gender, weight, duration of medical history, baseline platelet level, bleeding score, and previous medication of each patient group were analyzed separately ( Table 1 ). The results showed that all patient indicators in each group were normally distributed, and the *p* -values of the H test or chi-squared test were not statistically different among the three groups.

**Table 1:** Patient demographics and baseline characteristics (mean ± SD, %)

Characteristics	Corticosteroids (65 cases)	IVIg (69 cases)	IVIg and corticosteroids (71 cases)	*p* -Value (H or chi-square test)
Age (y)	42.8 ± 16.9	41.5 ± 13.19	38.8 ± 14.6	0.209
Sex, *n* (%)				
Male	34 (52.31)	30 (43.48)	37 (52.11)	0.785
Female	31 (47.69)	39 (56.52)	34 (47.89)	0.799
Weight (kg)	71.4 ± 11.4	69.6 ± 10.5	68.1 ± 13.1	0.179
Duration of thrombocytopenia (mo)	56 ± 19	61 ± 68	77 ± 84	0.073
Baseline platelet count (× 10 ^9^ /L)	10 ± 7	11 ± 7	9 ± 7	0.058
< 10 × 10 ^9^ /L, *n* (%)	30 (46.15)	33 (47.8)	51 (71.8)	0.204
10–30 × 10 ^9^ /L, *n* (%)	35 (53.85)	36 (52.17)	20 (28.2)	0.093
Bleeding (WHO bleeding scale grade 1–4), *n* (%)				
0	15 (23.08)	18 (26.09)	15 (21.13)	0.860
1	42 (64.62)	45 (65.21)	53 (74.65)	0.828
2	8 (12.30)	6 (8.70)	3 (4.23)	0.287
Previous therapies, *n* (%)				
Corticosteroids	34 (52.31)	40 (57.97)	29 (40.85)	0.486
IVIg	15 (23.08)	30 (46.48)	23 (32.39)	0.205
rhTPO/TPO receptor agonists	9 (13.85)	19 (27.54)	10 (14.08)	0.157
Cyclosporine	2 (3.08)	4 (5.80)	4 (5.63)	0.737
Danazol	13 (20.00)	7 (10.14)	8 (11.27)	0.297
Rituximab	3 (4.62)	2 (2.90)	3 (4.23)	0.873
Vincristine	3 (4.62)	0 (0)	2 (2.82)	0.231

Abbreviations: IVIg, intravenous immunoglobulin; rhTPO, recombinant human thrombopoietin; SD, standard deviation; WHO, World Health Organization.

### Efficacy Evaluation

#### Platelet Count and Response Rate Statistics of Patients in Each Group after Treatment

**Fig. 1:**
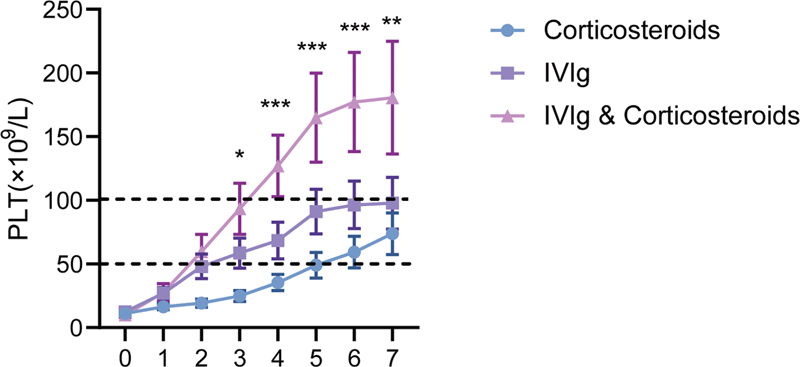
Curves of platelet counts in each group during the course of treatment. The platelet count of the combination group was significantly higher than that of the corticosteroids group from day 1 to day 7, and platelet counts were consistently significantly higher in the intravenous immunoglobulin (IVIg) group from day 3 to day 7. Compared with the corticosteroids group, the platelet count of the IVIg group showed a statistically significant difference from day 1 to day 6, and the difference disappeared with the increase of platelet count in the corticosteroids group on day 7 ( *p* = 0.08) ( *t* -test, * *p* < 0.05, ** *p* < 0.01, *** *p* < 0.001).

**Fig. 2:**
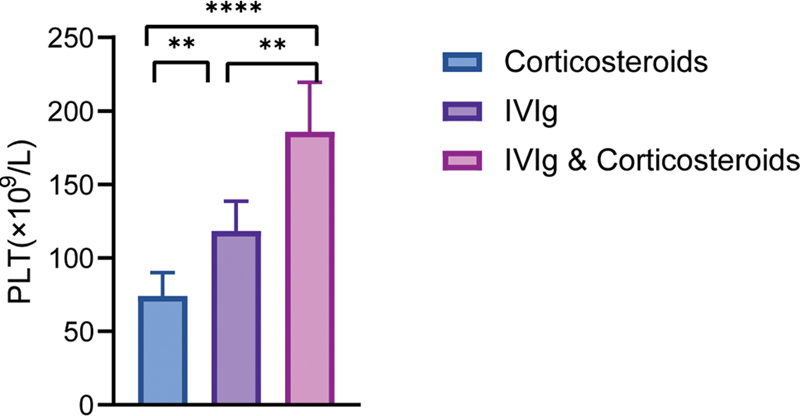
The PLT _max_ of each group during the course of treatment. Mean with 95% confidence intervals of PLT _max_ of patients in each group during the course of treatment ( *t* -test, * *p* < 0.05, ** *p* < 0.01, *** *p* < 0.001).

From the platelet counts of patients in each group within 7 days of treatment ( Figs. 1 and 2 ), it was evident that the recovery rate and PLT _max_ in the combination group were significantly higher than those in the corticosteroids and IVIg groups from day 3 to day 7. The platelet count of the IVIg group was significantly higher than that of the corticosteroids group from day 1 to day 6, and the platelet count of the corticosteroids group gradually reached its peak on day 7, after which the statistical difference between the two groups disappeared. In terms of the onset time and PLT _max_ after treatment, the efficacy could be ranked in descending order as follows: combination group, IVIg group, and corticosteroids group.

The proportion of patients who achieved CR after 7 days of treatment ( Table 2 ) was significantly higher in the combination group than in the corticosteroids alone group. However, there was no significant difference between the combination group and the IVIg alone group ( *p* = 0.08), and there was no significant difference between the corticosteroids alone group and the IVIg group ( *p* = 0.08). Additionally, there was no statistically significant difference in the proportion of partial response (PR) among the three groups. The CR rate of platelet count in the combination group was the highest and significantly higher than that in the corticosteroids group. The CR rate in the IVIg group was between the two groups, but there was no significant difference between the two groups. Furthermore, there was no statistically significant difference in PR levels after 7 days of treatment among the three groups. Therefore, we further evaluated the efficacy of treatment in the three groups by using the proportion of platelet counts reaching 30 × 10 ^9^ /L, 50 × 10 ^9^ /L, and 100 × 10 ^9^ /L ( Table 3 ). There was no significant difference in the proportion of platelet counts reaching 30 × 10 ^9^ /L among the three groups. The proportion of platelet counts reaching 50 × 10 ^9^ /L in the combination group was higher than that in the IVIg group and the corticosteroids group, but there was no statistical difference. The percentage of platelet counts reaching 100 × 10 ^9^ /L in the combination group was significantly higher than that in the glucocorticoid group, and the IVIg group was in between the two groups, but there was no significant difference between the combination group and the glucocorticoid group. These findings suggested that combination therapy of first-line drugs could improve the platelet counts of 50 × 10 ^9^ /L and 100 × 10 ^9^ /L within 7 days in more patients, and the efficacy was better than that of the two monotherapy groups.

**Table 2:** The number and percentage of patients achieving CR and R after 7 days of treatment in each group ( *n* , %)

Group	CR *n* (%)	R *n* (%)
Corticosteroids	15 (23.08)	48 (73.85)
IVIg	30 (43.48)	57 (82.61)
IVIg and corticosteroids	51 (71.83) a	59 (83.10)

Abbreviations: CR, complete response; IVIg, intravenous immunoglobulin; R, response.

a Chi-square test was used to compare the percentage of CR between the corticosteroids group and the combination group, *p* -value < 0.01.

**Table 3:** The number and percentage of patients whose platelets reaching 30 × 10 ^9^ /L, 50 × 10 ^9^ /L, and 100 × 10 ^9^ /L in each group on the 7th day after treatment ( *n* , %)

Group	≥ 30 × 10 ^9^ /L	≥ 50 × 10 ^9^ /L	≥ 100 × 10 ^9^ /L
Corticosteroids	50 (76.92)	37 (56.92)	15 (23.08)
IVIg	59 (85.51)	47 (68.12)	30 (43.48)
IVIg and corticosteroids	62 (87.32)	58 (81.69)	51 (71.83) a

Abbreviation: IVIg, intravenous immunoglobulin.

a Chi-square test was used to test the percentage of patients with platelet counts ≥ 100 × 109/L in the corticosteroids group and the combination group, *p* -value < 0.01.

#### Statistics of the Onset Time of Treatment

**Fig. 3:**
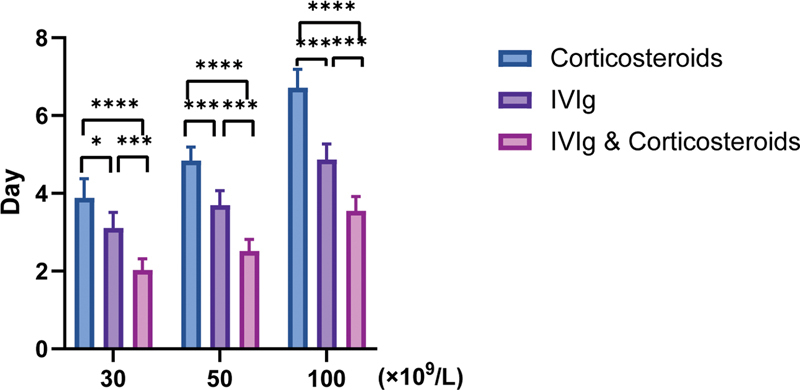
The treatment days corresponding to the platelet count reaching 30 × 10 ^9^ /L, 50 × 10 ^9^ /L, and 100 × 10 ^9^ /L for the first time. We calculated the mean with 95% confidence intervals for the number of days in which patients achieved platelet counts of 30 × 10 ^9^ /L, 50 × 10 ^9^ /L, and 100 × 10 ^9^ /L for the first time in each group. Patients who did not reach it were not included in the statistics ( *t* -test, * *p* < 0.05, ** *p* < 0.01, *** *p* < 0.001).

**Fig. 4:**
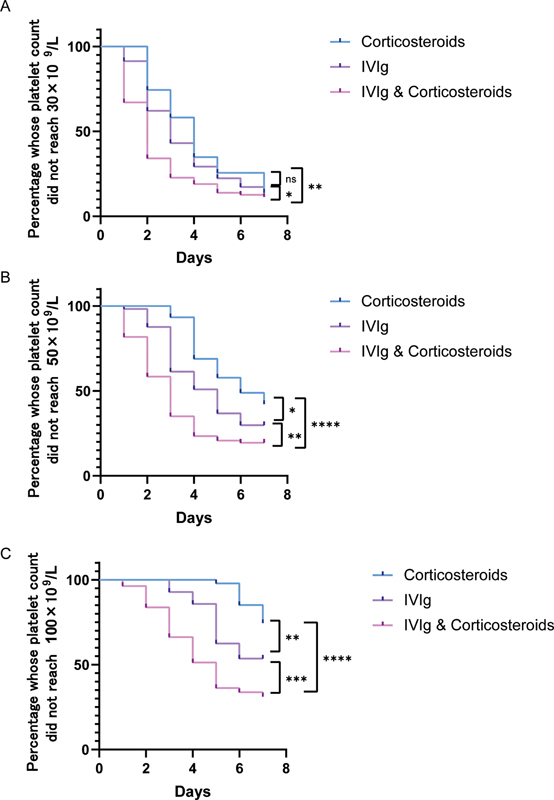
The percentage of patients in each group whose platelet count did not reach 30 × 10 ^9^ /L ( **A** ), 50 × 10 ^9^ /L ( **B** ), and 100 × 10 ^9^ /L ( **C** ) per day. The Kaplan–Meier method was used to calculate the number of patients with platelet counts reaching 30 × 10 ^9^ /L, 50 × 10 ^9^ /L, and 100 × 10 ^9^ /L every day, and the percentage curve of each group was drawn. The differences between groups were compared using the log-rank test (* *p* < 0.05, ** *p* < 0.01, *** *p* < 0.001, **** *p* < 0.0001).

Compared with the single corticosteroids group and the IVIg group, the combination group demonstrated a significantly shorter time to reach platelet counts of 30 × 10 ^9^ /L, 50 × 10 ^9^ /L, and 100 × 10 ^9^ /L for the first time, and the IVIg group achieved these platelet counts significantly faster than the corticosteroids group ( Fig. 3 ). This finding suggested that the combination group had the fastest onset of effect, followed by the IVIg group, and the corticosteroids group was the slowest. Furthermore, the proportion of patients in the combination group with platelet counts reaching 30 × 10 ^9^ /L, 50 × 10 ^9^ /L, and 100 × 10 ^9^ /L per day was significantly higher than that in the IVIg group and the corticosteroids group. The proportion of patients in the IVIg group with platelet counts reaching 50 × 10 ^9^ /L and 100 × 10 ^9^ /L per day was significantly higher than that in the corticosteroids group, but there was no significant difference in the proportion of patients with platelet counts reaching 30 × 10 ^9^ /L ( Fig. 4A–C ). Overall, these findings suggested that combination therapy had a faster onset of action and better improvement in platelet count than the two monotherapy groups. Although the proportion of patients with daily platelet counts reaching 30 × 10 ^9^ /L was similar in the IVIg group and corticosteroids group, there were significant differences in the proportion of patients with daily platelet counts reaching 50 × 10 ^9^ /L and 100 × 10 ^9^ /L, suggesting that IVIg and corticosteroids had a similar remission rate in the treatment of recurrent ITP. However, the improvement of platelet count in the IVIg group was significantly greater than that in the corticosteroids group.

#### Statistics of AEs

The AEs were generally mild and had a similar incidence and severity across the three groups. Less than 10% of patients reported AEs ( Tables 4 and 5 ). All AEs noted in this study have been previously reported.

**Table 4:** AEs during treatment

AE, *n* (%)	Corticosteroids	IVIg	IVIg and corticosteroids	*p* -Value (chi-square test)
Fatigue	4 (6.15)	5 (7.25)	6 (8.45)	0.892
Headache	0	3 (4.35)	0	0.056
Pyrexia	0	3 (4.35)	2 (2.82)	0.271
Palpitation	1 (1.54)	0	0	0.345
Insomnia	3 (4.62)	0	1 (1.41)	0.156
Hypertension	1 (1.54)	0	1 (1.41)	0.602
Hyperglycemia	2 (3.08)	0	1 (1.41)	0.344
Upper respiratory tract infection (URTI)	5 (7.69)	2 (2.90)	6 (8.45)	0.389
Urinary tract infections	2 (3.08)	0	1 (1.41)	0.344
Hypokalemia	4 (6.15)	2 (2.90)	5 (7.04)	0.555
Alanine aminotransferase increased	1 (1.54)	1 (1.45)	4 (5.63)	0.271
Aspartate aminotransferase increased	1 (1.54)	1 (1.45)	2 (2.82)	0.815
Bilirubin increased	0	2 (2.90)	2 (2.82)	0.398

Abbreviations: AE, adverse event; IVIg, intravenous immunoglobulin.

**Table 5:** Drug-related AEs during treatment

AE, *n* (%)	Corticosteroids	IVIg	IVIg and corticosteroids	*p* -Value (chi-square test)
Pyrexia	0	3 (4.35)	1 (1.41)	0.190
Hypertension	1 (1.54)	0	0	0.345
Hyperglycemia	2 (3.08)	0	0	0.121
Alanine aminotransferase increased	1 (1.54)	1 (1.45)	3 (4.23)	0.502
Aspartate aminotransferase increased	1 (1.54)	1 (1.45)	2 (2.82)	0.815
Bilirubin increased	0	2 (2.90)	1 (1.41)	0.387

Abbreviations: AE, adverse event; IVIg, intravenous immunoglobulin.

In the corticosteroids group, the most common AEs were upper respiratory tract infection (URTI) (7.69%), fatigue (6.15%), hypokalemia (6.15%), insomnia (4.62%), hyperglycemia (3.08%), urinary tract infections (3.08%), palpitation (1.54%), hypertension (1.54%), increased AST (1.54%), and increased ALT (1.54%). In the IVIg group, the most common AEs were fatigue (7.25%), headache (4.35%), pyrexia (4.35%), URTI (2.90%), hypokalemia (2.90%), increased bilirubin (2.90%), increased AST (1.45%), and increased ALT (1.45%). In the combination group, the most common AEs were fatigue (8.45%), URTI (8.45%), hypokalemia (7.04%), increased ALT (5.63%), pyrexia (2.82%), increased AST (2.82%), increased bilirubin (2.82%), insomnia (1.41%), hypertension (1.41%), hyperglycemia (1.41%), and urinary tract infections (1.41%).

Regarding drug-related AEs, in the corticosteroids group, they were hyperglycemia (3.08%) and hypertension (1.54%), increased ALT (1.54%), and increased AST (1.54%). In the IVIg group, they were pyrexia (4.35%), increased bilirubin (2.90%), increased ALT (1.45%), and increased AST (1.45%). And in the combination group, they were increased ALT (4.32%), increased AST (2.82%), pyrexia (1.41%), and increased bilirubin (1.41%). No significant difference in AEs was observed among the three treatment groups.

## Discussion

Corticosteroids and IVIg are the primary treatment options for ITP due to their mechanisms of action, which reduce platelet recognition by specific antibodies, 3 improve myeloid-derived suppressor cells, 4 5 upregulate regulatory T cells, and decrease platelet clearance. Usually, clinical treatment for ITP involves selecting one of these drugs. While previous research has focused on the emergency treatment of ITP and ITP during pregnancy, 6 7 8 few studies have examined the treatment of recurrent ITP in adults. Therefore, our study retrospectively reviewed clinical cases of persistent or chronically recurrent adult ITP patients from multiple centers and compared the efficacy of three treatment regimens in improving platelet count. Our goal was to provide an important reference for clinical treatment of this patient population.

Previous literature has suggested that the CR rate of newly diagnosed ITP patients treated with corticosteroids alone is 60%, 9 while the CR rate of high-dose dexamethasone treatment has no significant difference (81–85%). 10 11 However, in the present study, the CR rate of recurrent persistent and chronic ITP patients treated with corticosteroids again was only 23.08%. The CR rate of the combination group was 71.83%, which was significantly higher than that of the corticosteroids alone group and was not inferior to that of TPO-RA (71.8%) 12 13 and rituximab (23.7%). 14 As a result, corticosteroids should not be used alone in the retreatment of patients with persistent and chronic ITP who experience relapse. It should be combined with IVIg or changed to other drugs to achieve better efficacy. Although the proportion of the IVIg group was higher than that of the corticosteroids group, the difference was not statistically significant, which was inconsistent with previous reports. 15 This could be attributed to the small sample size of the study or the fact that the cases included in the study were limited to adult patients with relapsed ITP.

The CR rate of ITP patients treated with high-dose dexamethasone and conventional prednisone is reported to be 59%, with a median duration of CR of 14 days, 9 16 which is inconsistent with the results of the current study. It was possible that fewer patients in the corticosteroids group achieved CR after 7 days of treatment due to a shorter follow-up time. This might lead to a lower CR rate in the corticosteroids group and a shorter time to achieve the first 100 × 10 ^9^ /L platelet count (CR) during treatment.

The severity of bleeding in chronic ITP patients is negatively correlated with platelet count, 17 18 especially in patients with platelet count less than 20 × 10 ^9^ /L, where this relationship is more significant ( *r* = –0.345, *p* < 0.01). 19 Previous studies have defined the optimal threshold for bleeding as a platelet count of 20 × 10 ^9^ /L, 20 and platelet function can be better exerted when the platelet count is > 30 × 10 ^9^ /L, 21 thus improving bleeding symptoms. Therefore, when the patient's platelet count increases to 30 × 10 ^9^ /L, it is basically equivalent to the relief of bleeding symptoms. This study calculated the number of days after treatment when the platelet count reached 30 × 109/L in patients. The results showed that the combination therapy group had the shortest time to alleviate bleeding symptoms, followed by the IVIg group, and the corticosteroids group had the longest time.

After 1 week of treatment, there was no significant difference in the response rate among the three groups, suggesting that the response rate of corticosteroids, IVIg, or glucocorticoids combined with IVIg in the treatment of persistent and chronic recurrent ITP was still 73 to 83%. This was similar to the response rate of TPO-RA drugs in the treatment of newly diagnosed or chronic ITP (74–94%). 22 23 24

The average onset time for the combination group was 2 days, while it was 3 days for the IVIg group and 4 days for the corticosteroids group. The average number of days required to reach platelet counts of 30 × 10 ^9^ /L, 50 × 10 ^9^ /L, and 100 × 10 ^9^ /L in the combination group was 2, 3, and 4 days after treatment, respectively. These numbers were significantly higher than those for the monotherapy groups, indicating significant differences in onset speed and efficacy among the three groups.

Previous studies have demonstrated that combining corticosteroids with IVIg or other medications can produce the most favorable outcomes in the shortest possible time for emergency treatment of ITP or ITP during pregnancy. 6 25 26 Our results suggested that combination therapy with first-line drugs also had advantages in terms of onset time and efficacy for patients with persistent and chronic relapsed ITP. This type of therapy is especially suitable for patients with thrombocytopenia and bleeding symptoms that do not reach emergency treatment. Combination therapy could effectively increase platelet counts in a short time and achieve hemostasis while reducing the physical and economic burden of large doses of medication on patients. It also reduced the frequency of platelet transfusions.

In addition, there was no significant difference in the proportion of platelet counts reaching 30 × 10 ^9^ /L between the IVIg and corticosteroids groups. However, the proportions of patients achieving platelet counts of 50 × 10 ^9^ /L and 100 × 10 ^9^ /L were significantly different between the two groups, suggesting that the response rates to IVIg and corticosteroids were similar. However, the onset time of the IVIg group was faster than that of the corticosteroid group, and the efficacy of IVIg was better than that of corticosteroids alone in the treatment of adult patients with relapsed ITP. Therefore, IVIg should be preferred as the first-line monotherapy for adult patients with relapsed ITP.

Combined administration of IVIg and corticosteroids did not increase AEs during treatment while improving R and CR rates, reducing recurrence rates, and resulting in faster and more effective improvement of platelet counts. This approach also alleviated bleeding symptoms and anxiety in patients as soon as possible, reducing their economic burden. For patients who cannot tolerate or afford full-dose 5% IVIg treatment due to poor heart function, obesity, poverty, or other reasons, the combined application can shorten the treatment cycle while ensuring efficacy and reducing the body burden of patients. Achieving CR earlier can also shorten the course of treatment, avoiding the occurrence of infection, gastrointestinal ulcers, metabolic disorders, osteoporosis, and other treatment-related side effects.

With the development and progress of biomedical technology, more and more new drugs and targeted drugs provide clinical options for the treatment of ITP patients. However, there is no clear consensus on the optimal sequence of medications, leading to heterogeneity in the treatment of relapsed ITP. 27 Due to the side effects of long-term corticosteroid use and the difficulty in maintaining the efficacy of IVIg, many patients prefer using TPO-RA, rituximab, Syk inhibitors, and Fcγ receptor (FcγR) inhibitors when the disease recurs. 28 Approximately 80% of patients with persistent or chronic recurrent ITP use TPO-RA drugs, 76% choose rituximab, and 55% choose other types of immunosuppressants. In addition, the probability of using cortisol drugs for the first recurrence is approximately 75%, while the probability of using TPO-RA and rituximab for the second and third recurrences is increased to 57 and 52%, respectively. 27 When second-line drug treatment fails, the immune disorder mechanism in the patient's body becomes more complex, leading to an increased incidence of refractory ITP.

Currently, most first-line drug treatment studies focus on the efficacy and safety of newly diagnosed ITP patients and strategy innovation. 9 Real-world clinical studies and cross-sectional comparisons of treatment options for patients with relapsed ITP are limited. In this study, we retrospectively analyzed the clinical efficacy and safety of three first-line drugs used alone or in combination within 1 week. It was clear that IVIg combined with glucocorticoid had a faster onset time and better clinical efficacy than monotherapy in the treatment of recurrent ITP in adults. This study provided reliable clinical evidence and reference for the first-line drug treatment of adult recurrent ITP. However, since this study is a retrospective study, the level of evidence is lower than that of prospective studies, and clinicians still need to choose the appropriate treatment plan based on their clinical experience and patients' conditions. We look forward to more prospective studies for these kinds of patients from clinical research centers across the country.

## Conclusion

Our study has shown that the combination of IVIg and corticosteroids is a more efficient and rapid treatment for relapsed ITP in adults compared with the use of either therapy alone. The safety profile of the combination therapy was similar to that of monotherapy. These results offer valuable clinical evidence and serve as a useful reference for the use of first-line combination therapy in the treatment of relapsed ITP in adult patients.
